# Modulating Rheological Properties via Non-Cross-Linked Phase in Biphasic Hyaluronic Acid Fillers

**DOI:** 10.1021/acsomega.5c02674

**Published:** 2025-09-04

**Authors:** Orhan Gokalp Buyukuysal, Zeynep Caglar, Alkin Ozgen, Halil Murat Aydin

**Affiliations:** † Institute of Science, Bioengineering Division, 37515 Hacettepe University, Beytepe, Ankara 06800, Turkey; ‡ Centre for Bioengineering, Hacettepe University, Beytepe, Ankara 06800, Turkey

## Abstract

Hyaluronic acid (HA) based soft tissue augmentation materials are high-value-added products that have been used as filling materials in many areas such as the chin, face, and hands for many years. In our study, the synthesis methodology that utilizes a less toxic cross-linker to obtain stiffer gels with a high storage modulus (G′) leads to much safer dermal fillers. For this purpose, citric acid (CA) was used as a green cross-linker. By altering the amount of CA, a series of non-cross-linked gels were prepared and reconstituted with a size-reduced cross-linked phase to prepare biphasic hyaluronic acid fillers with different mechanical and rheological characteristics. The effect of steam sterilization on the biphasic gels’ physical properties was examined. No toxic waste was encountered after steam sterilization in the biphasic gels. Non-cross-linked HA fractions with a concentration of 23 mg/mL and containing 4% (w/w), 6% (w/w), 8% (w/w), 10% (w/w), and 12% (w/w) amounts of CA were prepared. 1,4-Butanediol diglycidyl ether (BDDE) was used at 6% (w/w) concentration for cross-linked hyaluronic acid (CL-HA) gel synthesis. The BP-E-10-S sample was determined as the optimal group. Rheological results of the BP-E-10-S sample such as elastic modulus (G′, Pa), loss modulus (G″, Pa), complex viscosity (η*), and phase angle (○) were measured 388.76 ± 13.91, 88.89 ± 6.22, 12.69 ± 0.47, and 12.86 ± 0.45, respectively. Cell viability was evaluated by an MTT assay using L929 cells. *In vivo* biocompatibility analysis of the BP-E-10-S group was performed following the ISO 10993 standards. Histopathological evaluations were evaluated and concluded to be nonirritant and biocompatible with an analysis score of 1.92 following protocol and the evaluation criterion of ISO 10993-6:2016. In addition, a patent was obtained for the formulations.

## Graphical abstract

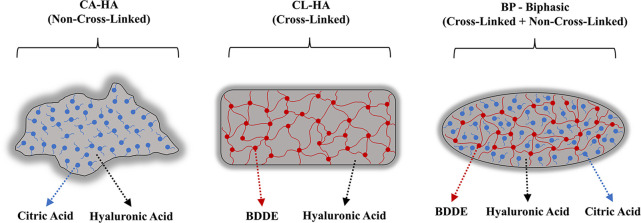

## 1 Introduction

The impacts of years of facial movement, gravity, and sun exposure all start to show on an aging face. Wrinkles on the face are a result of the skin tissues aging in a youthful way. Soft tissue fillers can help fill these wrinkles by providing augmentation/reconstruction of tissue cells, resulting in a smoother and more youthful appearance.^1^

Dermal fillers for soft tissue are among the most popular nonsurgical esthetic procedures for facial rejuvenation. The ideal filler is not permanent, should last a long time, have few adverse effects, do not require allergy testing, be simple to use, not hurt during the procedure, and be affordable. Hence, the durability of dermal fillers has been a source of concern.

Hyaluronic acid (HA) is a high molecular weight biopolysaccharide.^1,2^ It occurs naturally in human tissues, found mainly in the extracellular matrix, but recent studies have shown that it also occurs intracellularly.^3–5^ HA is involved in several biological processes, such as cell separation, supramolecular assembly of proteoglycans in the extracellular matrix, and control of mitosis, migration, tissue hydration, and water transport. Moreover, it preserves the elastoviscosity of liquid connective tissues such as the vitreous fluid in the eyes, joints, and skin. HA binds water and lubricates the body’s working parts, including the muscles and joints. It can be employed as a great moisturizer in skin augmentation and reconstruction products due to its viscosity and tissue compatibility. HA is one of the most hydrophilic molecules in nature.^5–8^

In physiological pH, HA is a polyanionic polymer. Because of its high charge, it has a large water-binding capacity. Although HA has a good biocompatibility and affinity for water molecules, it is soluble in physiological media and is rapidly removed. Therefore, to provide tissue reconstruction for skin wrinkles, HA is cross-linked to improve mechanical properties and residence time at the implant site.^6^

Cross-linkers form a polymer network and join the HA polymer chains together. HA gel acts as a single unit, forming a chemical and physical barrier against enzymatic and free radical destruction. Enzymes and free radicals are limited in their ability to break down larger parts of the chains at a time due to the multilinked nature of the gel network. In addition, cross-linking slows down the enzymatic degradation kinetics and produces a longer-lasting product.^9,10^

1,4-Butanediol diglycidyl ether (BDDE) has become the industrial standard cross-linker due to its stability, biodegradability, and long-term safety. BDDE reacts with the hydroxyl sites in HA chains and plays an effective role in slowing down the enzymatic and free radical degradation of dermal fillers when injected into the skin.^1,2,11^

BDDE undergoes degradation through hydrolysis. Cross-linked HA fillers should contain <2 ppm unreacted BDDE in the final product according to FDA guidelines.^12,13^ Owing to the absence of reactive epoxide groups, any of the other chemical states of BDDE are unlikely to lead to additional toxicity risks. Most of the epoxide groups are opened under the alkaline cross-linking reaction conditions (pH > 7). So, it can either hydrolyze to alcohol or connect to HA via an ether bond. Even in neutral environments, there is a good chance that minute amounts of leftover cross-linker in the final product will hydrolyze. For BDDE cross-linked HA fillers, a substantial amount of biocompatibility data is available in addition to over 15 years of clinical safety data.^14–17^

The degree of cross-linking among fillers with equivalent HA concentrations is correlated with viscosity and hardness. The amount of cross-linked HA and the degree of cross-linking inside the gel are significant factors that influence how much the fillers are cross-linked. Lower concentrations of HA will produce softer hydrogels. Non-cross-linked HA is added to cross-linked HA fillers to increase mechanical properties such as viscosity and elastic modulus to form biphasic gels. Overly high viscosity makes filler injection challenging. The viscosity is lowered by adding some free HA, making it easier to inject the filler.^15,18^ Another important feature of the biphasic gels is their long degradation time compared with monophasic gels. Also, the heterogenic structure of the biphasic gels provides ease of personalization for applications.^19^

Citric acid (CA) is classified by the Food and Drug Administration (FDA) as a safe, inexpensive, and UV-resistant monomer listed in the GRAS category.^20^ CA does not chemically accumulate and is metabolized without causing any significant side effects, even at very high concentrations. It has an advanced safety profile since it is a byproduct of the Krebs cycle. Moreover, CA is considered a green cross-linker due to its three carboxylic acid and one hydroxide functional groups. The presence of the three carboxylic acid groups in the structure of CA provides the possibility for cross-linking of various biopolymers via the ester or amidation reactions between the hydroxyl or amine groups of biopolymers and carboxylic groups of CA.^21^ The cross-linking mechanism of CA is dependent on covalent bonds formed by the chemical reaction between HA and the cross-linker. In addition, it includes hydrogen bonds and electrostatic physical cross-links defined as van der Waals interaction.^22^ Utilizing these bonding mechanisms of CA causes an increase in the gel structural stability and mechanical properties.^23,24^ Other important aspects of CA usage for gels and soft tissue augmentation would be its biocompatibility, accessibility, and multifunctionality. The study is focused on creating HA-based biphasic gels that contain non-cross-linked and cross-linked fractions. The non-cross-linked fractions were obtained by modifying HA with different amounts of CA. Simultaneously, the cross-linked fractions were obtained using BDDE as a chemical cross-linker. Then these fractions were combined with different ratios to produce biphasic HA gels. Rheological and physicochemical properties were investigated before and after steam sterilization.

## 2 Materials and Methods

First, CA-HA fractions containing CA at concentrations of 4, 6, 8, 10, and 12% (w/v) were synthesized. Subsequently, cross-linked HA gels (CL-HA) in microparticulate form were produced. Afterward, biphasic gel synthesis was carried out using CL-HA and CA-HA fractions to obtain the final product, which is one of the main goals of the study. CA-HA fractions were reconstituted with CL-HA at 5, 10, and 15% (w/w) to obtain 15 different products. Finally, the effect of steam sterilization on biphasic gels was examined by comparing the analysis results before and after sterilization.

### 2.1 Preparation of Citric Acid-Modified Non-Cross-Linked Fractions (CA-HA)

To obtain CA-modified cross-linked fractions, injection-pure HA (Bloomberg) was dispersed into injection-pure water (WFI) at a concentration of 23 mg/mL and mixed with an analogue mechanical mixer. After the dissolution was completed, CA (Sigma, Aldrich) was added at the rates of 4, 6, 8, 10, and 12% by weight (w/w), respectively, and mixed for 24 h. The resulting CA-HA fractions were named CA-HA-A, CA-HA-B, CA-HA-C, CA-HA-D, and CA-HA-E, respectively. NaOH (Sigma-Aldrich) and HCl (Isolab, Germany) buffers were used to adjust the pH range (6.5–7.5).^25^ Samples were stored at +4 °C for further use.

### 2.2 Microparticulate Cross-Linked HA (CL-HA) Gel Preparation

To produce HA gels in cross-linked particulate form, concentrated HA solution was prepared in NaOH, and 6% (w/w) BDDE (Sigma-Aldrich) was added and cross-linked at 52–53 °C for 3 h. After cross-linking, the gel was neutralized with HCl and buffered with PBS to a final concentration of 23 mg/mL. Afterward, dialysis was performed to remove unbound BDDE.^25^

### 2.3 Biphasic Gel Reconstitution

For the final product, CA-HA and CL-HA were reconstituted at room temperature with an analog mechanical mixer at 180 rpm to contain 5, 10, and 15% (w/w) non-cross-linked CA-HA fractions by weight. Table 1 provides detailed information about the biphasic gel formulations. BP stands for biphasic. A, B, C, D, and E stand for CA fractions with CA content of 4, 6, 8, 10, and 12% (w/w), respectively. 5, 10, and 15% (w/w) stand for the percentage of non-cross-linked materials that biphasic gels contain.

**Table 1:** Biphasic Gel Formulations

**% CA-HA content**	**group**	**CA-HA ratio** (w/w)	**CL-HA ratio** (w/w)
**5% (w/w)**	**BP-A-5**	with 4% CA - non-cross-linked	with 6% BDDE - cross-linked
	**BP-B-5**	with 6% CA - non-cross-linked	with 6% BDDE - cross-linked
	**BP-C-5**	with 8% CA - non-cross-linked	with 6% BDDE - cross-linked
	**BP-D-5**	with 10% CA - non-cross-linked	with 6% BDDE - cross-linked
	**BP-E-5**	with 12% CA - non-cross-linked	with 6% BDDE - cross-linked
**10% (w/w)**	**BP-A-10**	with 4% CA - non-cross-linked	with 6% BDDE - cross-linked
	**BP-B-10**	with 6% CA - non-cross-linked	with 6% BDDE - cross-linked
	**BP-C-10**	with 8% CA - non-cross-linked	with 6% BDDE - cross-linked
	**BP-D-10**	with 10% CA - non-cross-linked	with 6% BDDE - cross-linked
	**BP-E-10**	with 12% CA - non-cross-linked	with 6% BDDE - cross-linked
**15% (w/w)**	**BP-A-15**	with 4% CA - non-cross-linked	with 6% BDDE - cross-linked
	**BP-B-15**	with 6% CA - non-cross-linked	with 6% BDDE - cross-linked
	**BP-C-15**	with 8% CA - non-cross-linked	with 6% BDDE - cross-linked
	**BP-D-15**	with 10% CA - non-cross-linked	with 6% BDDE - cross-linked
	**BP-E-15**	with 12% CA - non-cross-linked	with 6% BDDE - cross-linked

### 2.4 Characterization of Gels before Sterilization

#### 2.4.1 Fourier Transform Infrared (FTIR) Spectroscopy

Structural analysis of CA-HA fractions and CL-HA group was carried out using attenuated total reflection Fourier transform infrared spectroscopy (ATR-FTIR, Agilent) with a resolution of 8 cm^–1^ in the wavelength range of 650–4000 cm^–1^. HA solution without CA was used as the control group for analysis.

#### 2.4.2 Rheology Measurements

A Kinexus (Malvern, UK) rheometer with a cone–plate geometry was used for rheological analysis. Strain sweep was carried out at a constraint frequency of 5 Hz within the range of 0.01–100% at 37 °C to determine the elastic modulus (G′), loss modulus (G″), complex viscosity (η*), and phase angle (^o^).

#### 2.4.3 Cohesivity Analysis

Cohesivity analysis was carried out by a drop-test method using CellScale Univert Biomaterial Tester (CellScale, Canada) under constant compression, by placing the samples in BD brand 1 cc syringes between load cells and flowing them at a speed of 7.5 mm/min until the moment of rupture.

#### 2.4.4 Particle Size Analysis

Particle size and distribution measurements were performed using the Mastersizer 3000 (Malvern, UK) particle analyzer, which calculates the length-to-length distribution of particles by measuring the angular intensity of the light scattered from the particles, using the principle of laser light scattering.

#### 2.4.5 Determination of Residual BDDE

Liquid chromatography-mass spectroscopy (LC-MS) analysis for BDDE residue in the gels was performed using Dionex Ultimate 3000 (Thermo Scientific).

#### 2.4.6 Citric Acid Release Analysis

To examine CA release in biphasic gels, samples taken on the first, seventh, and 14th days were analyzed using a UV–Vis spectrophotometer (Cary 60, Agilent). 0.1 M HCl was used as the release medium for the analysis. Samples were filtered with 45 μm filters at the designated times and stored at +4 °C until analysis. Concentration-dependent permeability values were obtained, and the release profile was created with the help of a calibration curve.

The CA maximum absorbance wavelength was determined by scanning at wavelengths of 230–800 nm. Subsequently, baseline analysis was performed using WFI at a wavelength of 230 nm. Finally, the samples taken on the first, fourth, and seventh days were measured at 230 nm wavelength. Measurements were repeated 3 times for each sample group. To obtain the calibration curve, HA fractions (23 mg/mL) with 1, 5, 10, 20, and 40% (w/w) CA addition were completely dissolved in 0.1 M HCl using a sonicator. Afterward, the maximum absorbance value was determined as 230 nm. The CA release calibration curve was created, and CA concentration calculations were made with the absorbance values.

### 2.5 Biphasic Gel Characterizations after Sterilization

To investigate the effect of steam sterilization on the mechanical and physical properties of the final products, poststerilization rheological analysis, cohesiveness analysis, and particle size analysis were completed.

#### 2.5.1 Material Sterilization

For the final product, a sterilization autoclave was used. Autoclave sterilization was carried out by exposing the biphasic gels to steam under high temperatures and pressure. The device parameters were studied in accordance with the conditions specified in ISO 17665-1, with a steam temperature of 121 °C and an application time of 15 min.

### 2.6 Degradation Analysis

Degradation analysis on the final product was done according to article 4.2.1, “accelerated degradation test” procedure, of “TS EN ISO 10993-13:2010 Biological evaluation of medical devices, Part 13: Identification and quantification of degradation products from polymeric medical devices,” as a service procurement from the Kırıkkale University Scientific and Technological Research Application and Research Center Directorate. Briefly, 10× PBS (SNP Biotechnology, Turkey) was diluted to 1× with deionized water. The sample, with a fixed weight of 1550 mg (w0), was placed in a cellulose filter for testing, in accordance with ASTM F1635 and TS EN ISO 10993-13:2010 standards. Then, the filter was placed into an impermeable test tube. 100 mL 1× PBS (pH: 7.4) was added to the tube and incubated at 70 °C. The final dry weight (w*r*) was measured at zeroth, first, ninth, 10th, and 18th days, respectively. The degradation rate was calculated as a function of weight loss using eq 1 in accordance with ASTM F1635 and TS EN ISO 10993-13:2010.

1 $$\mathrm{degradation}\;\mathrm{rate}\;\left(\%\right)=\frac{W_{0}-W_{r}}{W_{0}}\times 100$$

### 2.7 *In Vitro* Cytotoxicity Analysis

To determine the cytotoxic effect on the final product, indirect 3-(4,5-dimethylthiazol2-yl)-2,5-diphenyltetrazolium-bromide (MTT) analysis was performed in accordance with the ISO 10993-5 standard. For analysis, the final product was incubated in l-DMEM medium containing 10% FBS, 1% aa, and 1% l-glutamine at 37 °C in the presence of 5% CO_2_ using an incubator (Memmert, Germany) for 24 h. The extraction medium was filtered using a 0.45 μm filter and stored at +4 °C. L929 mouse fibroblast cell line was propagated using l-DMEM medium. Cells were seeded in a 24-well plate at 3 × 10^4^/well and placed in the incubator to proliferate for 24 h. Subsequently, the cells were fed with the previously obtained gel extracts at 1 mL/well and incubated for 24 h. After incubation, the extract was removed from the medium, and 60 μL of MTT (2.5 mg/mL, w/v) and 600 μL of l-DMEM were added and incubated for 3 h in a dark environment. Afterward, MTT-DMEM medium was removed from the wells, formazan crystals were dissolved with 405 μL of DMSO, and reading was carried out with a microplate reader (Biotek) at a wavelength of 570 nm, 200 μL/well in a 96-well plate. For analysis, the control group was incubated with l-DMEM medium containing 10% FBS, 1% aa, and 1% l-glutamine.

### 2.8 In Vivo Biocompatibility Analysis

*In vivo* biocompatibility analysis of the final product was carried out as a service procurement at TÜBİTAK Marmara Research Center as a 7 week gene implantation experiment. The implantation test was carried out according to “ISO 10993-6:2016 Biological evaluation of medical devices—Part 6: Tests for local effects after implantation,” “ISO 10993-2:2022 Biological evaluation of medical devices—Part 2: Animal welfare requirements,” and “ISO 10993-12:2021 Biological evaluation of medical devices—Part 12: Sample Preparation” protocols. TÜBİTAK Marmara Research Center is an establishment that has ethics approval for animal studies. Great care has been taken for the welfare of animals by following animal testing regulations and the 3R rule. Animals were euthanized within ethical rules and procedures after the study was completed.

Briefly, 3–4-month-old young adult Sprague Dawley rats, with weights of 250–300 g, were used for *in vivo* material testing. For this purpose, the BP-E-10-S material was injected into the lumbodorsal region as a subcutaneous implant. Silicone was used as the negative control group according to ISO 10993-12:2021 guidelines.

After 7 weeks, the test and control groups were sacrificed with cervical dislocation, and the local effects of the implants were identified and evaluated with histological staining. Ten tissue samples from the implant group, 10 tissue samples from the control group, and 3 tissue samples from the negative control were dissected. Then the tissues were fixated using 4% (w/v) paraformaldehyde followed by overnight tap water washing. The tissues were dehydrated with an ethanol series. After dehydration, the sample and control tissues were embedded in paraffin blocks and sectioned to 5 μm cross sections. The cross sections were stained with hematoxylin and eosin.

### 2.9 Statistical Analysis

The results are given by calculating standard deviation values (±SD). *n* = 3 samples were studied in all groups, and statistical analysis was performed using the one-way ANOVA test when comparing 3 or more groups. A comparison of 2 groups was made with a *t* test analysis (Tukey’s *t* test). The difference between groups was given as *p* > 0.5 nonsignificant, *p < 0.5, **p < 0.01, ***p < 0.001, and ****p < 0.0001.

## 3 Results

Within the scope of the study, (1) synthesis and characterization of CA-modified non-cross-linked HA fractions (CA-HA), (2) preparation and characterization of HA gel in particle forms (CL-HA), (3) obtaining and characterization of biphasic gels (BP Groups), (4) effects of steam sterilization on biphasic gels (BP-S Groups), and finally (5) the results of biodegradation and biocompatibility analyses of the final product are discussed.

### 3.1 Characterization of Citric Acid-Modified Non-Cross-Linked Fractions (CA-HA)

CA-HA fractions were synthesized using 4, 6, 8, 10, and 12% (w/w) CA and named as CA-HA-A, CA-HA-B, CA-HA-C, CA-HA-D, and CA-HA-E, respectively. The fractions were examined by using mechanical and physical characterization methods. Considering the FTIR results, CA and HA characteristic peaks were observed (SI Figure S1).

#### 3.1.1 Rheology Measurements

According to Figure 1, the control group has a high elastic modulus, due to its intrinsic properties and hydrogen bonding between HA chains and water.^26^ G′, G″, complex viscosity, and phase angle were found as 605 ± 2 Pa, 321.35 ± 1.15 Pa, 21.81 ± 0.075, and 27.98° ± 0.01, respectively (Figure 1). While high elastic modulus indicates that the control group is structurally stable, the phase angle, which is close to 45° implies liquid-like behavior.^27^ This shows that HA without any modification is not suitable for permanent dermal filler applications. In addition, the high elasticity of the control group is above the preferred range for dermal filler applications. Also, the non-cross-linked state of the control group prevents its use due to the intrabody enzymatic degradation of the non-cross-linked fraction taking 24–48 h.^28^ Moreover, it is seen that the addition of CA caused a decrease in G′ values (Figure 1 and Table 2).

**Figure 1:**
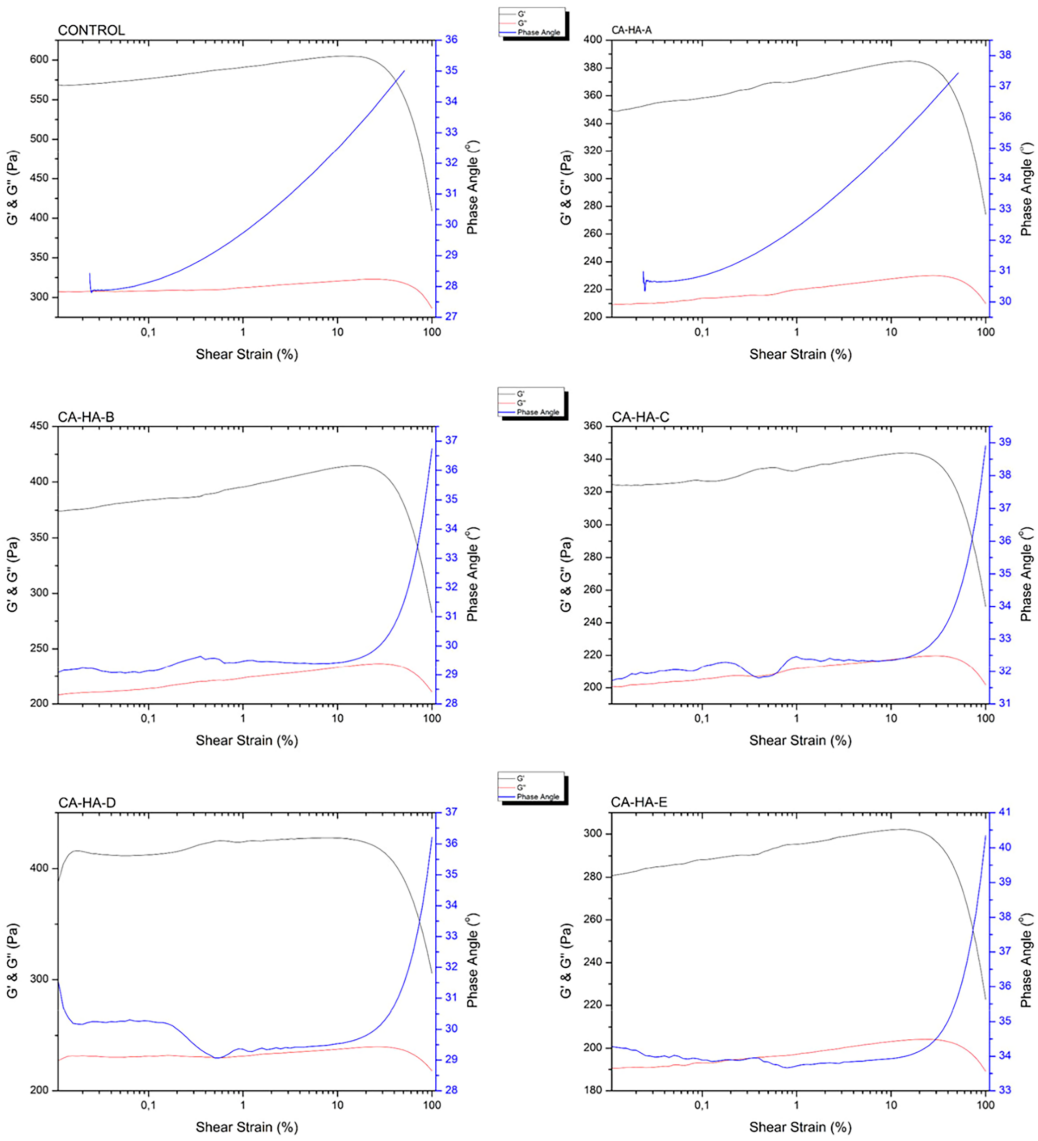
Rheology results of the CA-HA fractions.

**Table 2:** Rheology Measurements of CL-HA, CA-HA Fractions, and Control Group^a^

**sample**	**elastic modulus** (G′, Pa)	**loss modulus** (G″, Pa)	**complex viscosity** (η*)	**phase angle** (°)
**Control**	605 ± 1.63	321.35 ± 1.15	21.81 ± 0.07	27.98 ± 0.01
**CL-HA**	464 ± 8.27 (****)	74.93 ± 16.8 (****)	14.96 ± 0.3 (****)	9.15 ± 1.96 (****)
**CA-HA-A**	384.9 ± 6.5 (****)	228.85 ± 1.05 (****)	14.26 ± 0.195 (****)	30.74 ± 0.31 (**)
**CA-HA-B**	414.8 ± 10.1 (****)	235.15 ± 4.35 (****)	15.76 ± 0.34 (****)	29.55 ± 0.14 (ns)
**CA-HA-C**	343.8 ± 2.7 (****)	218.7 ± 0.9 (****)	12.97 ± 0.09 (****)	32.47 ± 0.09 (****)
**CA-HA-D**	426.35 ± 26.01 (****)	234 ± 2.7 (****)	15.57 ± 0.59 (****)	29.37 ± 1.57 (ns)
**CA-HA-E**	302.2 ± 0.6 (****)	203.8 ± 0.3 (****)	11.6 ± 0.02 (****)	34 ± 0.02 (****)

a (****P < 0.0001). One-way ANOVA was performed to compare multiple groups, and Tukey’s post hoc test was applied. *n*:3, *p* > 0.05 (ns), *p < 0.05; **p < 0.01; ***p < 0.001; ****p < 0.0001. Control: non-cross-linked hyaluronic acid.

An increase in phase angle was observed in CA-HA fractions compared with the control group. While there is a significant difference between the control group and CA-HA-A, CA-HA-C, and CA-HA-E at this point of increase, there is no significant difference between the control group and CA-HA-B and CA-HA-D.

Loss modulus (G″) gives information about the viscous property of the material. The higher the value, the more viscous behavior a material exhibits, and the more difficult it is to spread within the tissue.^29^ Since it is aimed to obtain a natural appearance in dermal fillers, the fraction is desired to be soft and has low viscosity.^30^ Finally, when the phase angle values of all fractions are examined, liquid-like behavior is observed.

The control group exhibits weak elastic behavior, considering all of its rheological properties. The sudden transition of the phase angle from the linear region to high values supports this.^10^ When the CA-added fractions were examined, considering the statistically significant decrease in the elastic modulus of these samples, it was evaluated that the CA-HA fractions were softer and more suitable for dermal filler applications. It can be seen that the elastic modulus was within the desired range for dermal fillers. The fact that G′ values are greater than G″ values in all groups supports this. In addition, the lower complex viscosity compared with the control group can be interpreted as the expression of viscoelastic behavior in CA fractions.^30^

#### 3.1.2 Cohesivity Analysis

Cohesiveness can be defined as the capacity of a gel not to separate. In other words, cohesivity is defined as the prevention of gel decomposition due to its high intermolecular affinity. Gel cohesiveness is an important parameter that ensures the preservation of gel integrity, supporting the structure of the natural tissue contour and eliminating tissue surface irregularities.^31^ Cohesivity results obtained by drop-weight analysis are given in SI Table S1.

An increase in cohesiveness is observed with the addition of CA (Figure 2); however, the cohesivity between the CA-added fractions does not have a statistically significant difference. Hence, increasing the amount of CA did not have a significant impact on the cohesiveness of CA-HA fractions. Also, high cohesion preserves the material integrity within the tissue; the material can support the tissue in the *in vivo* application area in the presence of intratissue mechanical stress.^32^

**Figure 2:**
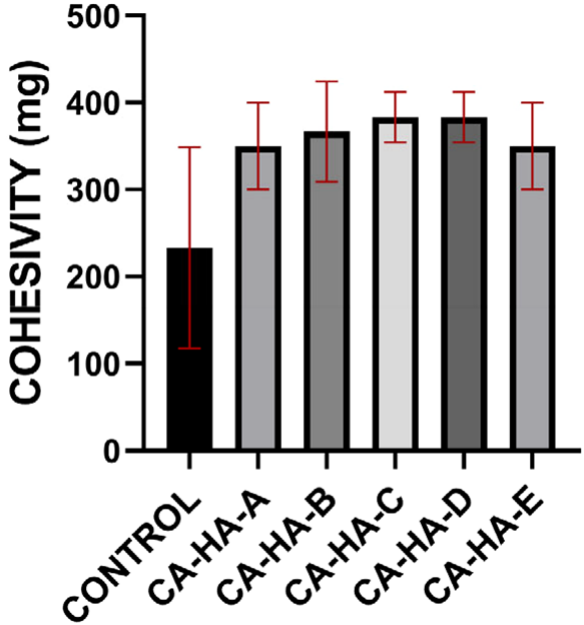
Cohesivity results for CA-HA fractions. One-way ANOVA was performed to compare multiple groups, and Tukey’s post hoc test was applied. *n*:3, *p* > 0.05 (ns), *p < 0.05; **p < 0.01; ***p < 0.001; ****p < 0.0001. Control: non-cross-linked hyaluronic acid.

### 3.2 Characterization of HA Gels in Cross-Linked Particulate Form (CL-HA)

Parameters such as the BDDE concentration, HA concentration, temperature, and reaction time were studied, and the optimum parameters were determined. The most efficient method was found to be using concentrated HA and 6% (w/w) BDDE with mixing at 52–53 °C for 3 h. Homogeneous cross-linking was achieved. Considering the FTIR results, HA characteristic peaks were observed (SI Figure S2).

#### 3.2.1 Rheological Measurements

G′, G″, complex viscosity, and phase angle were found as 464 ± 8.27 Pa, 74.93 ± 16.8 Pa, 14.96 ± 0.3, and 9.15° ± 1.96, respectively. When CL-HA rheological properties were compared with the control group, there is a statistically significant decrease, and a more stable gel structure is observed (Table 2 and Figure 3).

**Figure 3:**
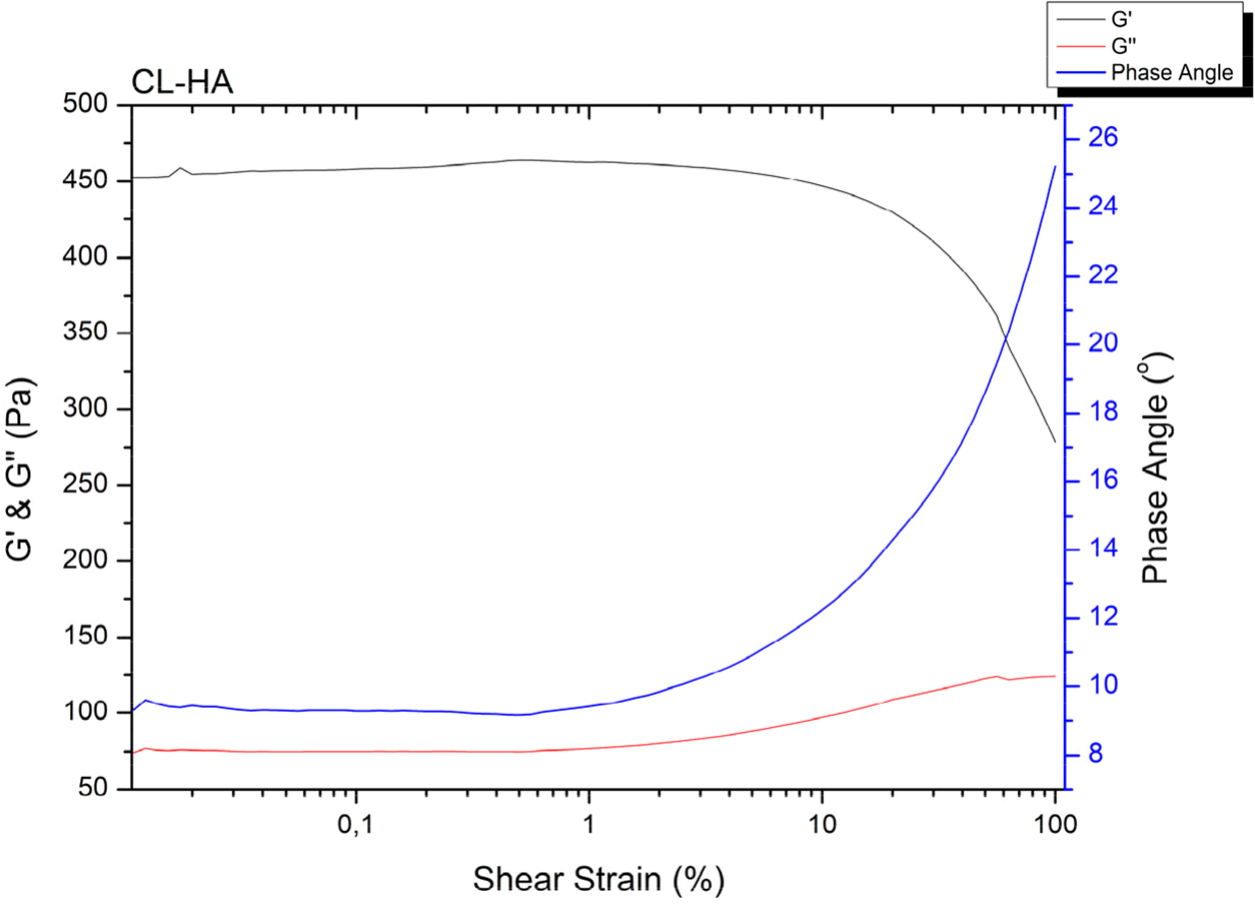
Rheology results of CL-HA fractions.

There is a decrease in the elastic modulus of CL-HA compared with the control group and CA-HA fractions (Table 2). The decrease in elastic modulus indicates that the CL-HA group has a softer structure.^33^ A homogenizer was used to obtain microparticulate form of CL-HA. This was seen as another factor causing the change in the elasticity of the material by mechanically breaking the polysaccharide chains in the material.^34^ Rheology results indicate that CL-HA exhibits better gel-like and viscoelastic behavior (Table 2). While the control group and CA-HA fractions show liquid-like behavior, the solid-like gel behavior of the CL-HA stands out.

As mentioned before, HA-based biomaterials are not suitable for *in vivo* use without any modification.^35^ CL-HA gels with increased chemical stability were obtained by cross-linking HA. Cross-linking reduces the water solubility of HA and enables gelation.^36^ This increases the efficiency of dermal filler applications by increasing both rheological viscoelastic behavior and water retention capacity.^37^ The most preferred cross-linking agent as a market standard is BBDE. In addition to BDDE slowing the enzymatic degradation of HA, the natural degradation of degradation products, 1,4-butanediol and glycerol, by the Krebs cycle or glycolysis, makes the cross-linker preferred. This is supported by the injectable HA gels on the market.^10^

#### 3.2.2 Particle Size Analysis

Particle sizes of CL-HA microgels were determined with laser diffraction particle size analysis. Microparticle size of CL-HA was determined as 1140 ± 81.8 μm (Figure 4).

**Figure 4:**
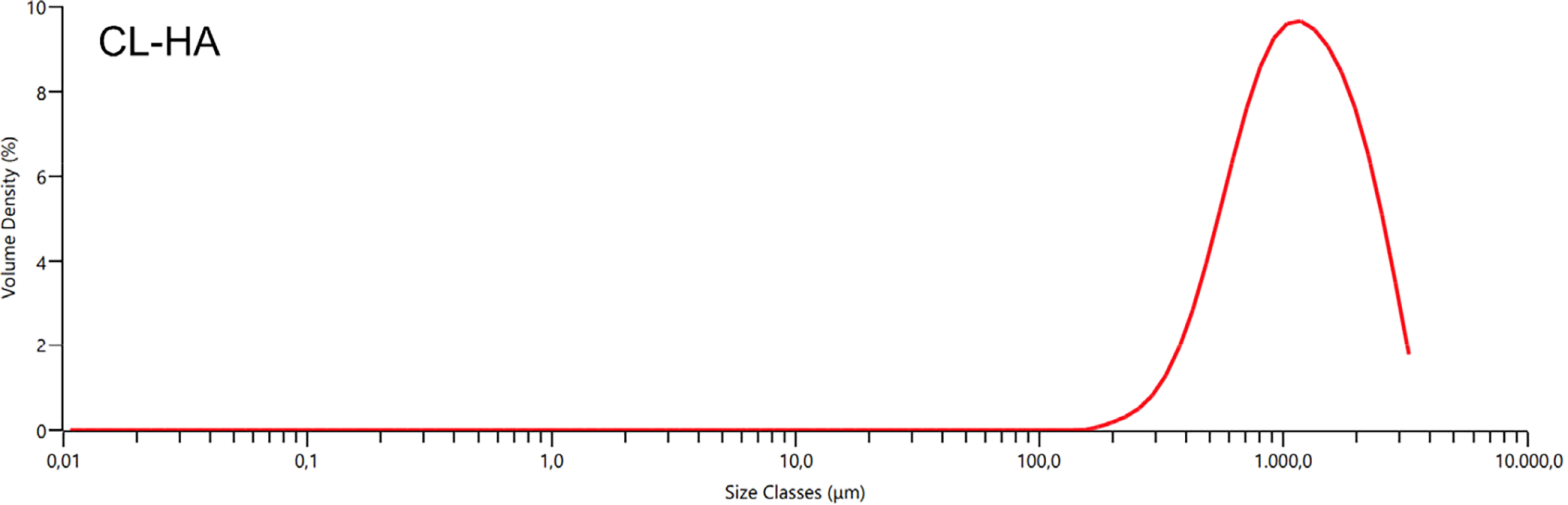
CL-HA size distribution.

CL-HA was fragmented with a homogenizer to obtain CL-HA microparticles. Cross-linking and fragmentation resulted in a softer gel structure as seen in the elastic module (Table 2) with a viscoelastic solid-like gel behavior. In terms of complex viscosity, microsized CL-HA and CA-HA fractions had similar results (Table 2), which were accepted as the indicator of fluidity of CL-HA microparticulate form. Since the flow property is directly affected by particle size, we can say particle size is an important feature for dermal fillers. For instance, HA fillers with small particle sizes are used in superficial applications, while HA fillers with large particle sizes are preferred in places where the dermis layer is thick and in deep dermal invasive applications.^5^ Also, in *in vivo* applications, it has been reported that the market leader Commercial Product 1, Commercial Product 2, and Commercial Product 3 have a 10, 11, and 15% reduction in particle size, respectively. Owing to hyaluronidase degradation, the largest decrease in particle size occurred in Commercial Product 3, which has the smallest initial particle size.^38^ Considering the effect of *in vivo* enzymatic degradation, CL-HA particle size is expected to be suitable for invasive deep dermal applications, and *in vivo* performance is expected to be more effective compared with other products. Particle size affects the rheological properties. Microparticulating the product with a homogenizer increases the fluidity of the product and reduces its elastic modulus.^39^

#### 3.2.3 Determination of Residual BDDE

Cross-linked HA fillers should contain <2 ppm unreacted BDDE in the final product according to FDA guidelines.^11,12^ Residual BDDE in the cross-linked CL-HA group was analyzed by liquid chromatography-mass spectroscopy (LC-MS). Solutions of 0.89 ± 0.06 g of CL-HA samples prepared as 10% (w/v) were analyzed. According to the analysis results, BDDE was found to be below the detectable threshold. The LC-MS calibration curve is in the Supporting Information (SI Figure S3).

### 3.3 Biphasic Gel Characterizations (BP Groups)

CA-HA fractions were reconstituted with synthesized CL-HA at 5, 10, and 15% (w/w), and biphasic gels were successfully obtained as the final products. Table 1 contains detailed information about the biphasic gels.

#### 3.3.1 Rheological Measurements

All groups exhibit solid-like behavior according to phase angle results (Table 3). According to G′, G″, and complex viscosity values, the viscoelastic feature attracts attention. It was found that there is no statistically significant difference between the elastic moduli and complex viscosities of the biphasic gel groups (Table 3).

**Table 3:** Rheology Results of the Final Products^a^

**sample**	**elastic modulus** (G′, Pa)	**loss modulus** (G″, Pa)	**complex viscosity** (η*)	**phase angle** (°)
**BP-A-5**	638.96 ± 54	83.28 ± 2.91	20.51 ± 1.69	7.5 ± 0.88
**BP-B-5**	633.71 ± 107.05	89.66 ± 0.9	20.38 ± 3.36	8.29 ± 1.46
**BP-C-5**	705.5 ± 119.05	87.71 ± 13.3	22.62 ± 3.81	712 ± 0.21
**BP-D-5**	611.15 ± 80.75	80.05 ± 2.15	19.62 ± 2.55	7.56 ± 0.79
**BP-E-5**	701.9 ± 144.91	93.93 ± 12.93	22.53 ± 4.62	7.74 ± 0.55
**BP-A-10**	761.16 ± 158	136.21 ± 30.6	24.61 ± 5.12	10.11 ± 0.18
**BP-B-10**	696.63 ± 154.05	147.35 ± 33.75	22.66 ± 5.02	11.93 ± 0.09
**BP-C-10**	597.4 ± 64.1	90.04 ± 9.1	22.56 ± 6.13	8.57 ± 0.05
**BP-D-10**	669.3 ± 47.3	96.52 ± 3.08	21.52 ± 1.51	8.22 ± 0.32
**BP-E-10**	597.41 ± 98.93	111.88 ± 15.39	19.25 ± 3.33	10.73 ± 0.39
**BP-A-15**	658.91 ± 168.5	111.63 ± 11.79	21.28 ± 5.35	9.98 ± 1.51
**BP-B-15**	698.81 ± 97.85	113.13 ± 24.45	22.54 ± 3.22	9.05 ± 0.96
**BP-C-15**	692.55 ± 117.45	133.68 ± 23.21	22.45 ± 3.81	10.91 ± 0.16
**BP-D-15**	617.06 ± 42.31	83.75 ± 5	19.82 ± 1.31	7.79 ± 0.98
**BP-E-15**	630.46 ± 98.1	112.41 ± 17.85	20.38 ± 3.17	10.1 ± 0.03

a One-way ANOVA was performed to compare multiple groups, and Tukey’s post hoc test was applied. *n*:3, *p* > 0.05 (ns), *p < 0.05; **p < 0.01; ***p < 0.001; ****p < 0.0001.

The rheological properties of biphasic gels are superior to those of CA-HA fractions and CL-HA (Tables 2 and 3). The increase in elastic modulus indicates that the biphasic gel is more stable and is stiffer than the CA-HA and CL-HA groups. It also shows that the solid-like behavior of biphasic gels is higher as the phase angle decreases.^33^ Biphasic gels, which have an advanced structure in terms of viscoelastic and elastic behavior, have the desired properties. The results have proven that the addition of CA in biphasic gels has an improving effect on rheological properties. Biphasic gels obtained by combining the CL-HA and CA-HA fractions have superior properties than both groups and have the properties of both fractions.^28^

#### 3.3.2 Cohesivity Analysis

The cohesive property of biphasic gels was assessed with drop-weigh analysis. The cohesivity results of biphasic gels are given in Figure 5.

**Figure 5:**
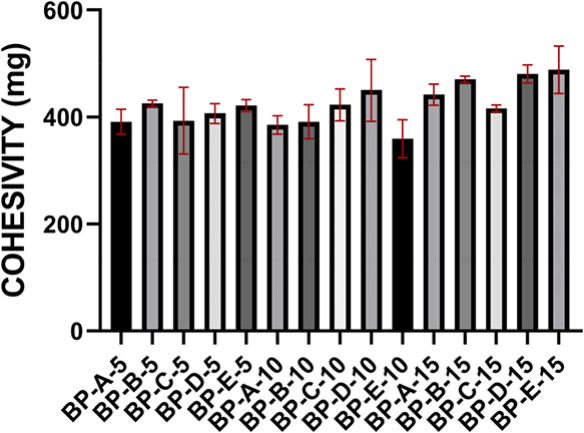
Cohesivity results of the BP groups. One-way ANOVA was performed to compare multiple groups, and Tukey’s post hoc test was applied. *n*:3, *p* > 0.05 (ns), *p < 0.05; **p < 0.01; ***p < 0.001; ****p < 0.0001. Control: non-cross-linked hyaluronic acid.

It is seen that there is no statistically significant difference between the cohesivity results (SI Table S2). High cohesiveness is observed in all groups. When the cohesivity results are evaluated with the rheological properties given in SI Table S2, structural stability attracts attention. It is thought that the resulting biphasic formulations have a high intermolecular affinity and will have a supportive effect on tissue integrity.^40^

#### 3.3.3 Citric Acid Release Analysis

The release profile of CA was studied by using UV–Vis spectrophotometry. The absorbance values at 230 nm wavelength were used to obtain the calibration curve (SI Figure S4). A calibration curve was used to determine the CA amounts in the extracts of biphasic gels (Table 4).

**Table 4:** CA Release Concentrations of Final Products on Day 14

**sample**	**citric acid concentration** (%)
**BP-A-5**	34.95
**BP-B-5**	not detected
**BP-C-5**	17.82
**BP-D-5**	22.06
**BP-E-5**	37.23
**BP-A-10**	26.82
**BP-B-10**	47.7
**BP-C-10**	not detected
**BP-D-10**	not detected
**BP-E-10**	7.86
**BP-A-15**	not detected
**BP-B-15**	not detected
**BP-C-15**	59.38
**BP-D-15**	45.58
**BP-E-15**	not detected

CA was not detected in the extracts of the final product groups BP-B-5, BP-C-10, BP-D-10, BP-A-15, BP-B-15, and BP-E-15. The results show that CA was removed from the biphasic gel structure after extraction at intervals of up to 14 days. It is known that CA is metabolized naturally in the Krebs cycle. For this reason, the fact that it was not detected in the 14th day extracts was considered as evidence that the release of CA was completed before 14 days.^41^ In addition, it has been accepted as an indicator that intrabody accumulation will not occur in *in vivo* application.

### 3.4 Biphasic Gel Characterizations after Sterilization

#### 3.4.1 Rheological Measurements

Elastic modulus (G′), loss modulus (G″), complex viscosity (η*), and phase angle (^o^) were obtained by rheology measurements of the final products. The obtained G′, G″, phase angle, and complex viscosity are given in the Supporting Information (SI Tables S3 and S4). The S suffix was used for steam-sterilized samples. There was a statistically significant decrease in G′ values in all groups. There was a loss of over 40% in G′ in all groups except BP-E-10. In addition, the increase in phase angle and decrease in complex viscosity (SI Tables S3 and S4) in all groups except BP-E-10 are considered as indicators that the gels show liquid-like behavior and structural stability decrement. A loss of more than 40% in G′ was accepted as an indication that the elastic and viscoelastic properties of the gels decrease significantly after sterilization.^42^ The rheological results of biphasic gels after sterilization are given in Figure 6.

**Figure 6:**
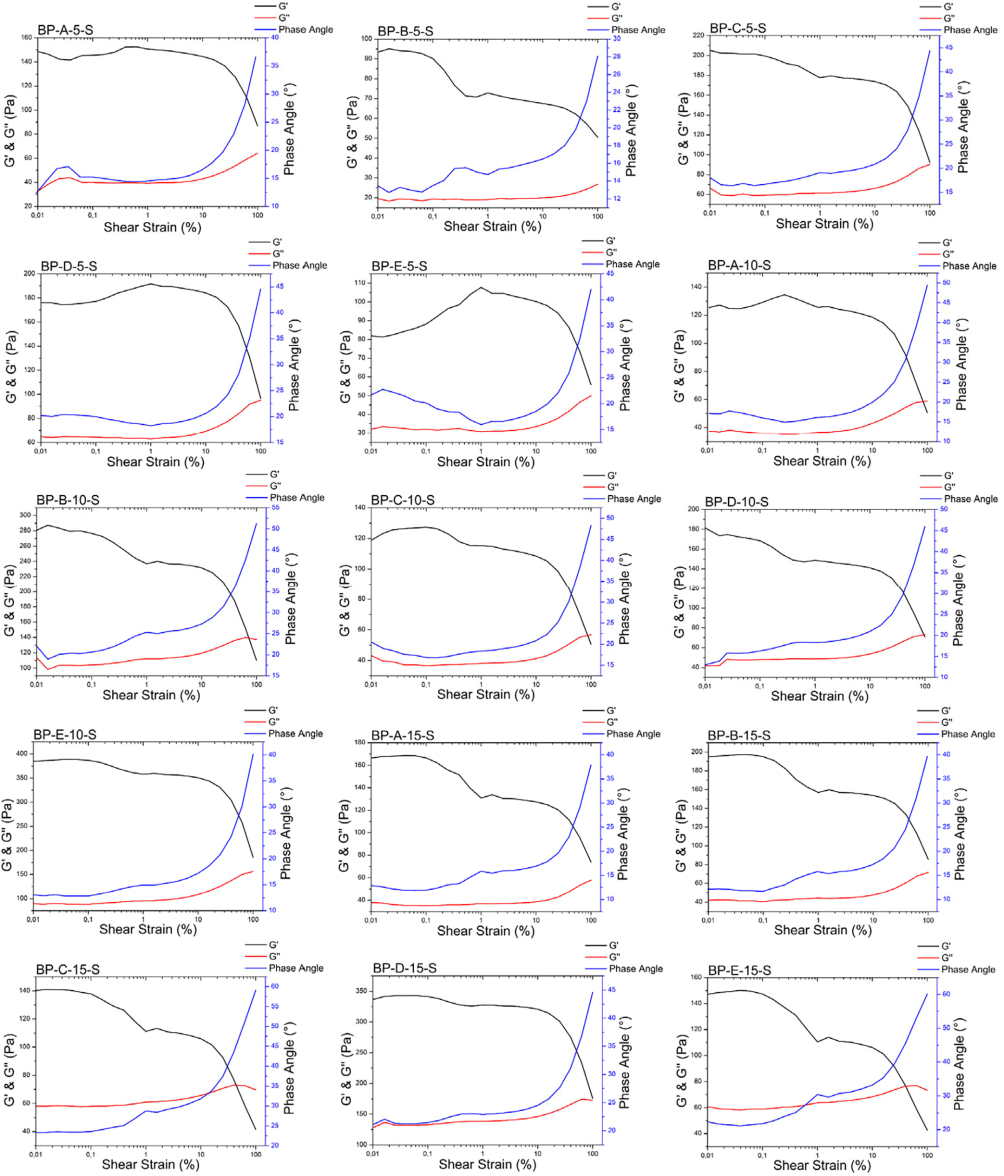
Rheology results for BP-S groups.

Based on G′ results, a statistically significant difference was found between BP-E-10-S and BP-A-5-S, BP-B-5-S, BP-C-5-S, BP-D-5-S, BP-E-5-S, BP-A-10-S, BP-B-10-S, BP-C-10-S, BP-D-10-S, BP-A-15-S, BP-B-15-S, BP-C-15-S, and BP-E-15-S (****P < 0.0001) and BP-D-15 group (**P < 0.01). BP-E-10-S was chosen as the ideal group, and other characterizations were carried out only with this group. There was no statistically significant difference in G″, complex viscosity, and phase angle between BP-E-10 and BP-E-10-S. Steam sterilization did not have a reducing effect on gel fluidity and preserved viscoelastic behavior (Table 5).

**Table 5:** Comparison of the BP-E-10 Group before and after Sterilization^a^

**sample**	**elastic modulus** (G′, Pa)	**loss modulus** (G″, Pa)	**complex viscosity** (η*)	**phase angle** (°)
**BP-E-10**	597.41 ± 98.93	111.88 ± 15.39	19.25 ± 3.33	10.73 ± 0.39
**BP-E-10-S**	388.76 ± 13.91	88.89 ± 6.22	12.69 ± 0.47	12.86 ± 0.45
**CL-HA**	464 ± 8.27	74.93 ± 16.8	14.96 ± 0.3	9.15 ± 1.96
**CL-HA-S**	312.6 ± 7.96	67.47 ± 0.97	10.18 ± 0.24	12.18 ± 0.42

a CL-HA-S was used for steam-sterilized CL-HA. One-way ANOVA was performed to compare multiple groups, and Tukey’s post hoc test was applied. *n*:3, *p* > 0.05 (ns), *p < 0.05; **p < 0.01; ***p < 0.001; ****p < 0.0001.

#### 3.4.2 Cohesivity Analysis

Cohesivity values of BP-E-10 and BP-E-10-S were calculated as 359.66 ± 35.52 and 230.33 ± 7.23 mg, respectively (Figure 7). A statistically significant decrease (**** *P* < 0.0001) of 35% was observed in cohesiveness results after sterilization of BP-E-10. Although there was a loss in cohesive structure, the decrease was within the acceptable range for dermal fillers.^16^

**Figure 7:**
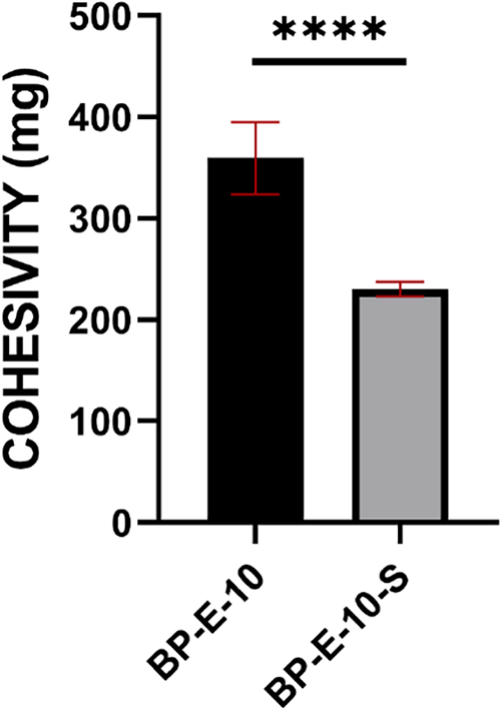
Cohesivity results of BP-E-10 and BP-E-10-S. One-way ANOVA was performed to compare multiple groups, and Tukey’s post hoc test was applied. *n*:3, *p* > 0.05 (ns), *p < 0.05; **p < 0.01; ***p < 0.001; ****p < 0.0001.

#### 3.4.3 Particle Size Analysis

To examine the effect of steam sterilization on particle size, CL-HA and BP-E-10 were sterilized and analyzed with the laser diffraction method (Figure 8). The particle sizes of CL-HA, CL-HA-S, and BP-E-10-S were 1140 ± 81.8, 1170 ± 27.5, and 1180 ± 34.1 μm, respectively. It was shown that steam sterilization and reconstruction did not affect the particle size.

**Figure 8:**
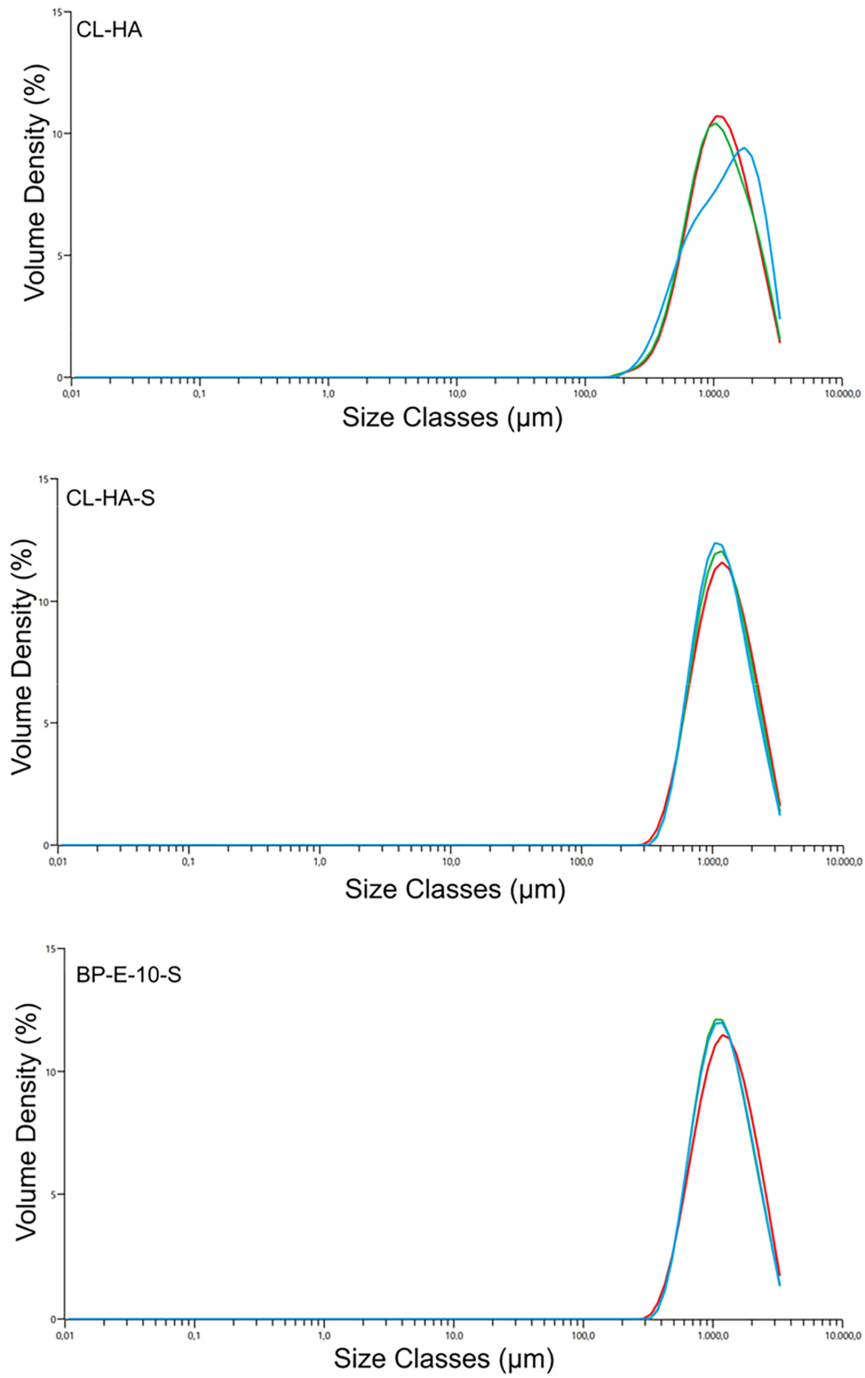
Laser diffraction results of CL-HA, CL-HA-S, and BP-E-10-S, respectively.

#### 3.4.4 Degradation Analysis

The degradation analysis of BP-E-10-S, which was the final product, was carried out in accordance with “TS EN ISO 10993-13:2010 Biological evaluation of medical devices—Part 13: Identification and quantification of degradation products from polymeric medical devices.” The degradation rate was assessed by calculating the weight loss of the sample as a function of time (Figure 9). The final product showed a stable degradation profile with a relatively fast degradation rate. Also, a degradation resistance time of more than 10 days was considered as an indicator of high stability.^43^ BP-E-10-S was considered as a successful product due to poststerilization rheological and cohesive properties.

**Figure 9:**
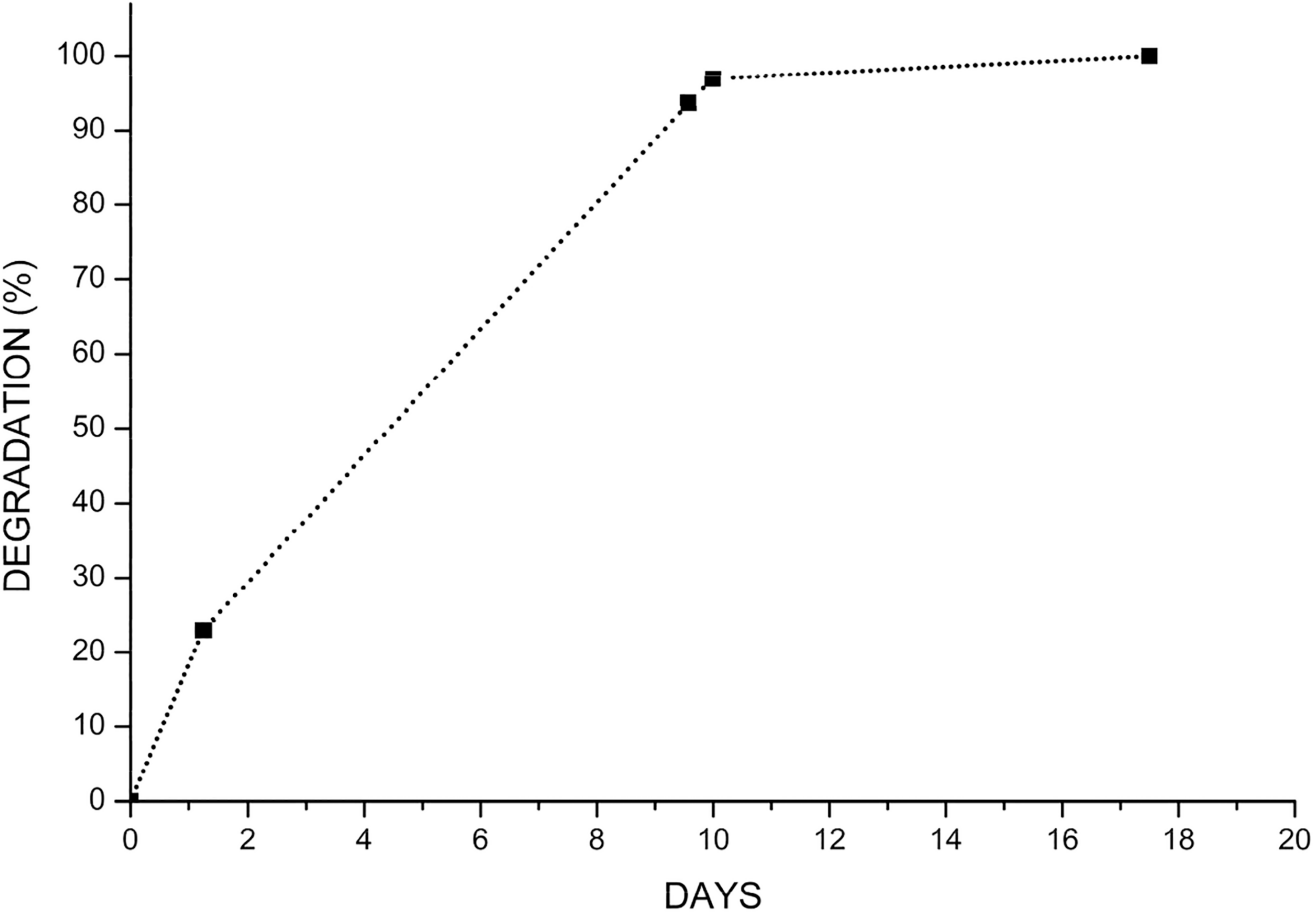
BP-E-10-S time-dependent degradation curve.

### 3.5 In Vitro and In Vivo Analysis

#### 3.5.1 In Vitro Cytotoxicity Analysis

BP-E-10-S cell viability was evaluated by MTT assay according to “TS EN ISO 10993-5:2009 Biological evaluation of medical devices—Part 5: Tests for *in vitro* cytotoxicity.” L929 cells incubated in l-DMEM growth medium were used as a control. Cell viability of BP-E-10-S gel was found to be 95% (Figure 10). No adverse effect on cell viability was detected.

**Figure 10:**
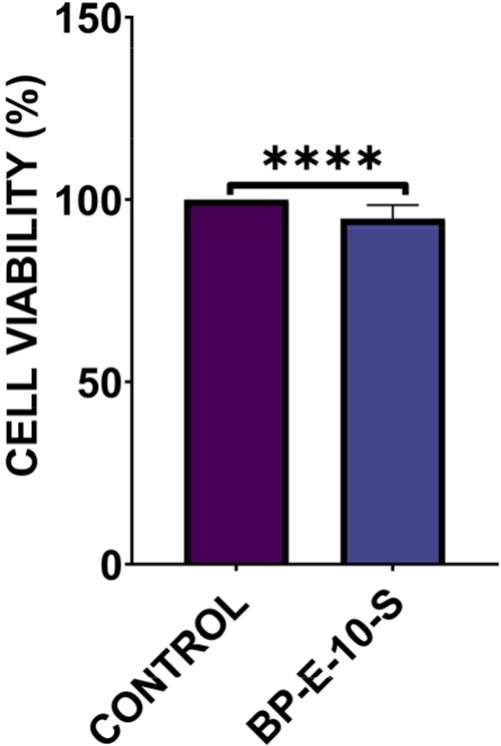
BP-E-10-S cytotoxicity analysis result. One-way ANOVA was performed to compare multiple groups, and Tukey’s post hoc test was applied. *n*:3, *p* > 0.05 (ns), *p < 0.05; **p < 0.01; ***p < 0.001; ****p < 0.0001.

#### 3.5.2 In Vivo Biocompatibility Analysis

*In vivo* biocompatibility of BP-E-10-S, which was determined as the final product, was performed in accordance with the TS EN ISO 10993-2:2022, 10993-6:2016, and 10993-12:2021 standards.

There were no necrotic or any other findings found at the inspection sites of the abdomen and thorax for the test and control groups after the cervical dislocation (Figure 11). The cell viability and tissue integrity of BP-E-10-S were evident when compared with the control group.^44^

**Figure 11:**
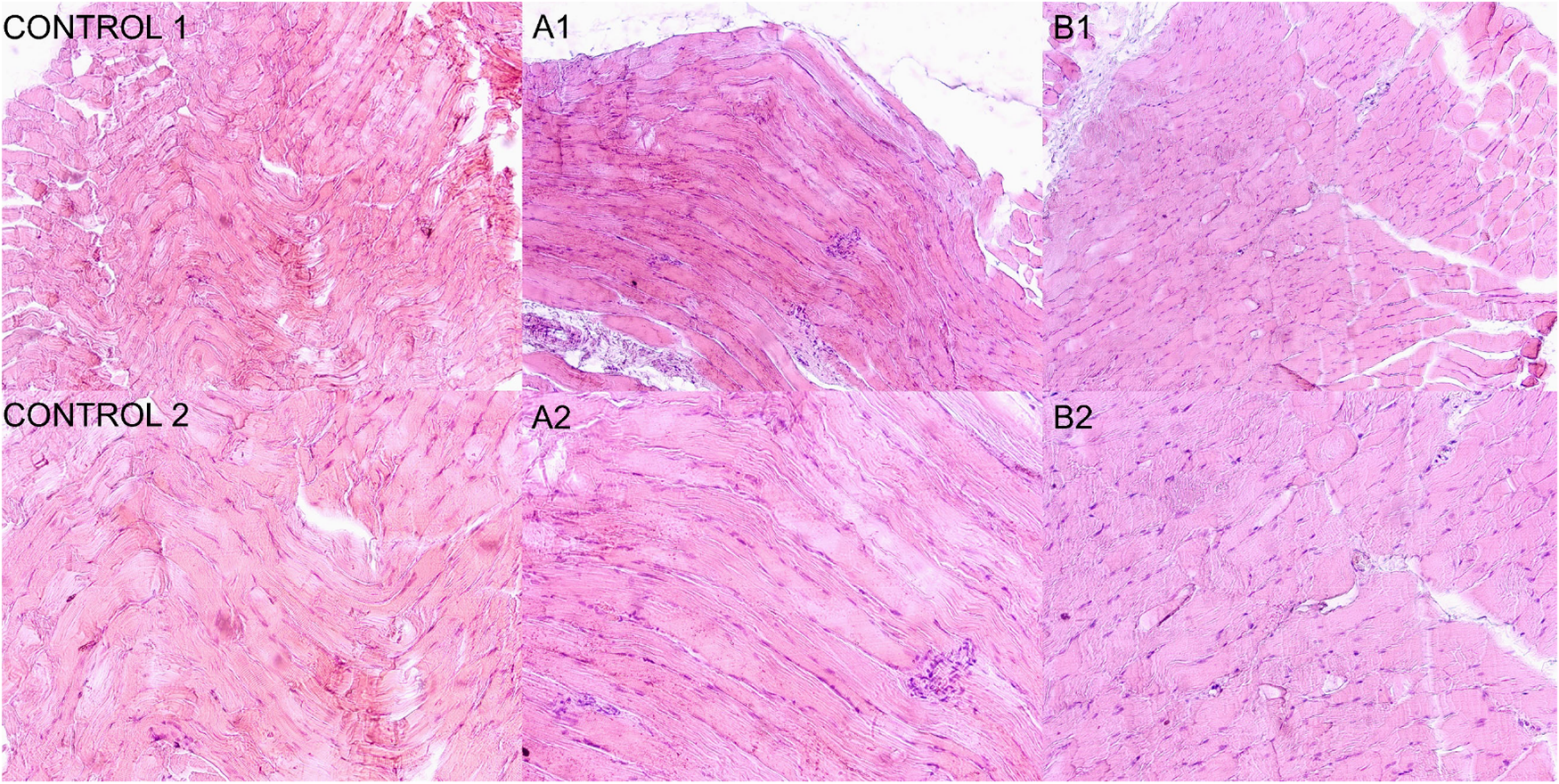
Histological evaluation of control and test groups. (Control 1) H&E staining of control group, 10× magnification. (Control 2) H&E staining of control group, 20× magnification. (A1) H&E staining of test group 1, 10× magnification. (A2) H&E staining of test group 1, 20× magnification. (B1) H&E staining of test group 2, 10× magnification. (B2) H&E staining of test group 2, 20× magnification.

Histopathological evaluations, given in Table 6, were performed, and the BP-E-10-S group as the final product did not show irritating properties. Plasma cells, giant cells, and necrosis were not observed at the implantation site (Figure 11). A macrophage and lymphocyte score below 1 was accepted as an indicator that there was no inflammatory response in the region.^45^ New vascularization and fat infiltration were not detected, and insignificant fibrosis (score of 0.75) was observed (Table 6). A score below 1 indicates that there is no significant connective tissue-induced solidification in the area.^46^ During the 7 week experiment, there was no inflammatory cell infiltration at the implant sites, and the product proved to be biocompatible with no irritant effect.

**Table 6:** BP-E-10-S Group Implantation Experiment Result Score Table

**test sample**	BP-E-10-S					
**implantation time**	7 weeks					
**control sample**	silicone					
**parameters**	**test sample**			control sample		
**the number of animals**	1001	1002	1003	1001	1002	1003
**inflammation**						
polymorphonuclear cells	0.00	0.00	0.00	0.00	0.00	0.00
lymphocytes	0.75	0.75	0.75	0.00	0.25	0.25
plasma cells	0.00	0.00	0.00	0.00	0.00	0.00
macrophages	0.25	0.00	0.25	0.00	0.00	0.00
giant cells	0.00	0.00	0.00	0.00	0.00	0.00
necrosis	0.00	0.00	0.00	0.00	0.00	0.00
**subtotal**	**2.00**	**1.50**	**2.00**	**0.00**	**0.50**	**0.50**
new vascularizations	0.00	0.25	0.25	0.00	0.00	0.25
fibrosis	0.75	0.75	0.75	0.50	0.75	0.00
lipid infiltration	0.00	0.00	0.00	0.00	0.00	0.00
**subtotal**	**0.75**	**1.00**	**1.00**	**0.50**	**1.75**	**0.25**
**total**	**2.75**	**2.50**	**3.0**	**0.50**	**1.25**	**0.75**
**group total**	**8.25**			**2.50**		
**mean** ^a^	**2.75 (−)**			**0.83 = >1.92**		
**traumatic necrosis**	0	0	0	0	0	0
**foreign rash**	0	0	0	0	0	0
**number of examined samples** ^b^	**4**	**4**	**4**	**4**	**4**	**4**
**sample score**	**2.75–0.83 = 1.92**					

a Irritant areas shown in the results are used. The negative difference must be zero.

b Irritant Histological Evaluation score indicates the examined implants mean score of the animal.

Histopathological analyses were carried out to evaluate clinical observations, gross pathology inspection, and implantation sites for the test material, control group, and negative control. The biocompatibility property of the BP-E-10-S group was determined according to the criteria given in “ISO 10993-6:2016 Biological evaluation of medical devices—Part 6: Tests for local effects after implantation.” The BP-E-10-S group was found to be nonirritant with an analysis score of 1.92 according to the protocol and the evaluation criterion of ISO 10993-6:2016.

### 3.6 General Discussion

High elastic modulus is accepted as an indicator for the non-cross-linked HA’s, which was the control group, structural stability despite the high phase angle value, which implies liquid-like behavior^27^ (Table 2). The weak mechanical and cohesive properties of the control group were supported by the sudden transition of the phase angle from the linear region to high values.^10^ These findings demonstrate that HA without any modification is not suitable for permanent dermal filler applications as the non-cross-linked state of the control group can be degraded within 24–48 h with intrabody enzymatic degradation.^28^ So, non-cross-linked HA fractions containing different amounts of CA with a concentration of 23 mg/mL were prepared. Increasing CA concentration increased the stability in CA-HA fractions and supported structural stability. In this context, different amounts of CA were encapsulated into the gel, acting as a green cross-linker and increasing the structural stability of the gel by interacting with the OH^–^ groups and secondary bonds in the HA structure.^47^ Since HA solution above isoelectric point causes electrostatic repulsion between the functional groups of HA, the intramolecular distance and mesh size increase, which allows CA to penetrate the 3D network of HA easily.^48^ As the amount of CA is increased in the polymeric network, the intramolecular and intermolecular interactions between CA and HA increase proportionally, resulting in highly stabilized CA-HA fractions, which can be proved by rheology analysis and G′ values^49^ (Figure 1). In terms of G′ evaluation, CA-HA fractions were softer and had less viscous behavior. In addition, the lower complex viscosity compared with the control group can be interpreted as the expression of viscoelastic behavior in CA fractions.^30^ It has also been reported that CA increases gel structural stability and has a positive effect on mechanical properties.^23,24^

The concentration of HA and CA has a direct impact on rheological properties and gel cohesiveness.^50^ Gel cohesiveness ensures the preservation of gel integrity, supporting the structure of the natural tissue contour and eliminating tissue surface irregularities.^31^ Since high cohesion preserves the material integrity within the tissue, it is thought that the material can support the tissue in the *in vivo* application area in the presence of intratissue mechanical stress^32^ (Figure 2). Cohesiveness and gel affinity were accepted as indicators for CA-HA fractions suitable for intradermal applications.^51^ So, in the light of these results, CA-HA fractions showed compatible rheologic and cohesive properties with the commercial dermal fillers.^52^

Subsequently, HA gels were synthesized to be used in biphasic gel synthesis. CL-HA group was cross-linked with BDDE, which is used in many commercial products.^53^ Although BDDE is generally used at 4% (w/w) concentration in literature, it was used at 6% (w/w) concentration for CL-HA synthesis within the scope of the project.^54^ The removal of unbound BDDE by extending the dialysis time was proven by LC-MS analysis, and the toxic effect was eliminated. HA chemical stability was ensured by chemical cross-linking, and a viscoelastic gel with solid-like gel behavior was obtained.^55^

The main purpose of the study was the synthesis of biphasic gels using CA-HA fractions and CL-HA. To this end, 15 different biphasic gels were synthesized. In all groups, there was an increase in G′ values compared with CL-HA and CA-HA fractions. This was accepted as an indicator of higher energy storage capacity and stiffness for biphasic gels. Additionally, phase angle and loss modulus were supportive indicators of higher stiffness.^55^ The varying CA-HA fraction ratios did not create a significant difference in elastic modulus and complex viscosity (Table 3). It is thought that increasing the CA concentration in the biphasic gel did not affect its resistance to deformation and flow continuity.^56^ Although the addition of CA improved the mechanical properties in biphasic products, increasing the CA concentration did not create a significant difference between biphasic gels (Table 3). Biphasic gels obtained by combining the CL-HA and CA-HA fractions have superior properties than both groups and have the properties of both fractions, which can also be seen in cohesivity results (Figure 5).^28^ It is thought that the resulting biphasic formulations have a high intermolecular affinity and will have a supportive effect on tissue integrity (Figure 5).^40^ The obtained values were compatible with the literature.^57,58^ We think the products had a profile that would maintain gel integrity, support the tissue, and provide the necessary surface smoothness in *in vivo* applications.^59^ Since higher G′ values indicate implementation for volumizing applications, we think these formulations could be used for such procedures. The viscoelastic behavior and cohesive nature of the formulations would support the tissue volume and induce cellular activity for tissue regenerative capacity.^60,61^

The intermolecular affinity of the final products was examined by drop-weight cohesivity analysis. The drop-weight method was used for its correlation with Sundaram’s dispersion method^31^ and repeatability. The formation of long strands during drop-weight analysis was accepted as a cohesive property for the gel, and all groups showed cohesive properties (Figures 2 and 5). The addition of CA increased the cohesivity of non-cross-linked fractions and biphasic formulations.^62^ It was observed that biphasic final products retained their cohesive properties compared with CA-HA fractions (Figures 2 and 5). The obtained values were compatible with the literature, and the products had a profile that would maintain gel integrity, support the tissue, and provide the necessary surface smoothness in *in vivo* applications.^57^ The release profile of CA in biphasic gels was studied, and it was observed that CA was not detected at the end of the 14th day in many groups. Hence, it was accepted as the evidence of nonaccumulation for CA for *in vivo* applications. As a result, the biphasic final products were determined to have superior properties and exhibit the properties of both cross-linked and non-cross-linked phases. One of the most preferred methods in the literature for gel sterilization is steam sterilization within the scope of the “TS EN ISO 17665:2024 Sterilization of health care products—Moist heat—Requirements for the development, validation and routine control of a sterilization process for medical devices.” Various sterilization techniques have been reported in the literature. Among these, autoclave sterilization, despite inducing some degree of deterioration in rheological properties, has been deemed acceptable in our study due to the formulation of the biphasic gel, which effectively mitigates such losses and preserves key functional properties within tolerable limits. As demonstrated in our study, while a reduction in rheological performance is observed, this anticipated effect was accounted for during formulation. The biphasic gel system, by virtue of its dual-phase architecture, retains advantageous features poststerilization, such as injectability, shear resistance, and appropriate viscoelastic behavior.

After steam sterilization, a loss of 10–40% in the mechanical properties of the final products was expected.^63^ The exposure to high temperature and pressure damages the cross-linked gel structure and secondary interactions resulting in structurally weakened gels.^64^ Although poststerilization rheological results of the biphasic gels showed a loss of more than 40% in G′ values in all groups except BP-E-10, the gels did not lose their viscoelastic behavior and showed solid-like gel behavior. In addition, it is thought that in the remaining 14 groups, the weak intermolecular affinity after reconstitution and the noninteraction between phases resulted in a high loss of mechanical properties after steam sterilization.^18^

The BP-E-10 group had a decrease of 34% in the elastic modulus. It was concluded that structural integrity was better compared with those of other groups. The fact that there is no statistically significant difference in loss modulus and phase angle before and after sterilization supports this. Besides, it is thought that its mechanical properties are better compared with those of Commercial Products 1 and 2, whose rheological analyses were carried out within the scope of preliminary studies. A higher elastic modulus is an indication that the gel would bear tissue volume and adapt to facial tissue morphology. Furthermore, the phase angle was higher than that of Commercial Product 1, supporting gel-like behavior. This was considered as an indication that its fluency was between Commercial Products 1 and 2.^1^

The effect of citric acid content in the BP-E-10 group was evident under both presterilization and poststerilization conditions. To better elucidate this effect, a comparative evaluation was conducted between CL-HA and BP-E-10, as well as their sterilized counterparts, CL-HA-S and BP-E-10-S. Rheological parameters of these four samples are presented in Table 5, including elastic modulus (G′), loss modulus (G″), and complex viscosity (η*). These results clearly indicate that the presence of the citric acid fraction confers improved mechanical stability and viscoelastic behavior to the biphasic hydrogel system. A similar trend was observed in the sterilized samples. Although a general reduction in rheological values was expected due to the sterilization process, BP-E-10-S still retained superior properties compared with CL-HA-S. These findings highlight the beneficial contribution of the citric acid-containing fraction to the overall rheological performance of the biphasic gel, both before and after sterilization, demonstrating its potential to enhance the mechanical resilience and structural integrity of the formulation.

In BP-E-10, a 35% decrease in cohesivity was observed after sterilization. Although there was a loss of cohesive structure, it is within the desired range for dermal fillers.^16^ The resulting product is cohesive and rheologically suitable for intradermal applications and has been successfully produced. Moreover, no cytotoxic effect was observed in the final product according to the ISO 10993-5 standard. The product was successfully steam-sterilized, and the filler properties were preserved to a high extent. Likewise, the fact that it showed a stable degradation curve in the degradation study is another indicator of its degradation resistance and stability. It is thought to decompose slower than Commercial Product 2.^43^

## 4 Conclusions

With this study, a biphasic hyaluronic acid filler was produced. In this context, it has been shown that CA can be used as an alternative cross-linker by modifying and characterizing HA with an environmentally friendly approach. At this point, a contribution has been made to investigate the effects of CA. In addition, the effects of CA usage on the gel structure were evaluated, and mechanical and structural examinations were carried out. It has been observed that CA increases both the HA structural stability and the biphasic gel structural stability. CA’s importance as an alternative modification tool for dermal fillers has been demonstrated, making an important contribution to the literature.

Fillers with small particle size are not suitable for use in deep dermal applications and are more appropriate for superficial dermis treatments.^65,66^ For example, Commercial Product 4 was indicated for deep dermal to superficial subcutaneous injections by the FDA in 2007. The largest fraction of gel particles for Commercial Product 4 is between 940 and 1090 μm.^67^ When fillers with a small particle size are intended for use in the deep dermis, higher amounts of BDDE are required to maintain structural integrity.

Our biphasic gel, formulated with citric acid, exhibits a larger particle size and provides an enhanced volumizing effect. Additionally, the gel demonstrates improved flowability upon injection due to the increased flexibility imparted by secondary intermolecular interactions. This approach enables the treatment of deep wrinkles using fillers that are less toxic, owing to the reduced need for BDDE, while maintaining both the particle size and the injectability.

The incorporation of citric acid reduced the requirement for high levels of BDDE, thereby minimizing the covalent bond content and avoiding excessive stiffness and potential toxicity. By producing a filler with a particle size of 1140 μm, we successfully decreased BDDE usage while ensuring its suitability for deep dermal application.

In biphasic final products, the change caused by steam sterilization in mechanical and cohesive properties was examined. No toxic waste or byproduct was encountered after steam sterilization. The tested formulations have shown that gels can be developed at different concentrations and compositions in the production of fillers for soft tissue engineering. Lastly, a patent was obtained for the formulations.

## Supplementary Material



## Notes

FTIR and cohesivity analyses of cross-linked and uncross-linked HA fractions, rheological measurements of biphasic gel fractions before and after sterilization, residual BDDE concentration, and the release profile of citric acid. The Supporting Information is available free of charge at https://pubs.acs.org/doi/10.1021/acsomega.5c02674.

FTIR and cohesivity analyses of cross-linked and uncross-linked HA fractions, rheological measurements of biphasic gel fractions before and after sterilization, residual BDDE concentration, and the release profile of citric acid (PDF)
