# Supplementary material for: Modulating Rheological Properties via Non-Cross-Linked Phase in Biphasic Hyaluronic Acid Fillers
Source: ACS Omega. 2025 Sep 4;10(36):40942–57. doi: 10.1021/acsomega.5c02674 (PMC12444562; doi:10.1021/acsomega.5c02674)
Supplement: Supplementary file 1 [file ao5c02674_si_001.pdf]

Supporting Information (SI) for

Modulating Rheological Properties via Non-crosslinked Phase in Biphasic Hyaluronic Acid Fillers

Orhan Gokalp Buyukuysal[1], Zeynep Caglar[1], Alkin Ozgen[1] and Halil Murat Aydin*[1, 2]

[1] Institute of Science, Bioengineering Division, Hacettepe University, Beytepe, Ankara, Turkey

[2] Centre for Bioengineering, Hacettepe University, Beytepe, Ankara, Turkey

*Corresponding Author:
Prof. Dr. Halil Murat AYDIN
E-mail: hmaydin@hacettepe.edu.tr
Tel: (+90) 312 297 78 00
Bioengineering Division and Centre for Bioengineering,
Hacettepe University, 06800, Ankara, Turkey

9 Pages

1. RESULTS AND DISCUSSION

1.1. Characterization of Citric Acid Modified Non-crosslinked Fractions (CA-HA)

1.1.1. Fourier Transform Infrared Spectroscopy (FTIR)

Figure S1 shows the spectrum of CA-HA fractions. The wide frequency band observed at the wavelength of 3283 cm^{-1} shows the O-H stretching inherent in the structure of HA[1,2]. The moderately sharp peak observed at a wavelength of 1636 cm^{-1} indicates the 1° amine (N-H) bending in the D-N-acetylglucose amin structure of HA[3]. The peaks observed in the range of 1030-1100 cm^{-1} are of C-O-C and C-OH stretching vibration origin and originate from the polysaccharide structure. The O-H stretching observed at a wavelength of 3287 cm^{-1} in the control group, at a wavelength of 3257 cm^{-1} in group A and at a wavelength of 3265 cm^{-1} in the other groups is attributed to the presence of CA. The specified peak shift supports the formation of secondary bonds between CA and HA[4].

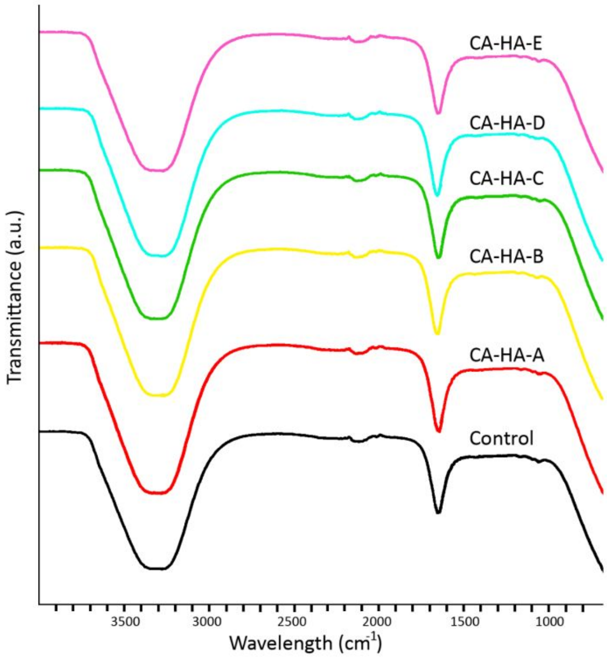

Figure S1. FTIR results of CA-HA group

Although there is a peak shift between the control group and CA-added fractions, this shift is not at a significant level, so FTIR spectra do not provide sufficient results to examine the difference in CA concentration between groups A, B, C, D and E. Considering this situation, comparative analysis of the results should be carried out by repeating the FTIR analysis with dry samples of the fractions to analyze whether there is any change in the results. Rheological measurements provide more clear evidence to prove the existence of CA. Peak shifts in the spectrum were evidence of the presence of CA.

3.1.2. Cohesivity Analysis

Table S1. Cohesivity results of CA-HA fractions.

Sample	CA Concentration (%w/w)	Cohesivity (mg)
Control	-	233.33 ± 115.47
CA-HA-A	%4	350 ± 50
CA-HA-B	%6	366.67 ± 57.74
CA-HA-C	%8	383.33 ± 28.87
CA-HA-D	%10	383.33 ± 28.87
CA-HA-E	%12	350 ± 50

1.2. Characterizations of HA Gels in Cross-linked Particulate Form (CL-HA)

1.2.1. Fourier Transform Infrared Spectroscopy (FTIR)

There was O-H stretching in the structure of HA at 3283 cm^{-1}. The sharp peak observed at a wavelength of 1636 cm^{-1} indicates the 1° amine (N-H) bend in the D-N-acetylglucose amin structure of HA. The peaks observed in the range of 1030-1100 cm^{-1} are of C-O-C and C-OH stretching vibration origin and originate from the polysaccharide structure. The peak shift of O-H stretching from 3283 cm^{-1} to 3321 cm^{-1} was accepted as the crosslinking between BDDE and epoxy groups (Fig. S1 and S2).

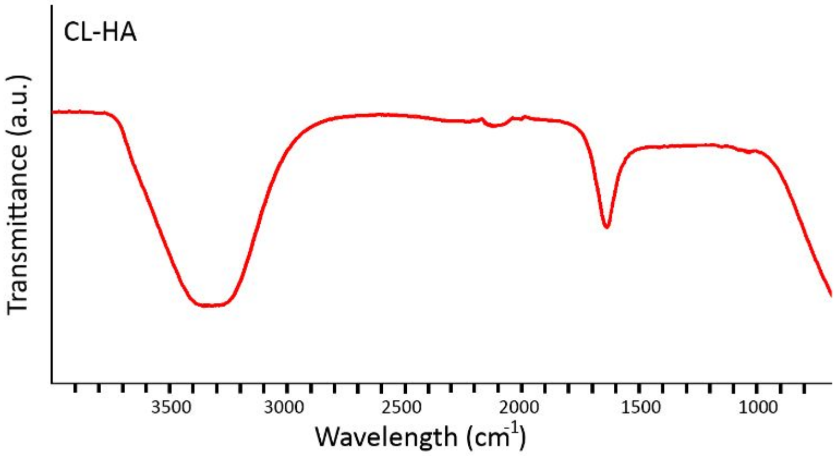

Figure S2. FTIR result of CL-HA

Although a peak shift was observed in the CL-HA group analysis, this shift does not reflect the change in intermolecular bonds to a significant level in FTIR measurements. Hence, the peak shift seen in the FTIR spectrum cannot be presented as evidence alone[2]. FTIR and rheology results were evaluated together to understand the presence of crosslinking. The decrease in loss modulus seen in the rheology supports the peak seen in the FTIR spectrums (Fig. S1 and S2)

and was presented as evidence of crosslinking.

1.2.2. Determination of Residual BDDE

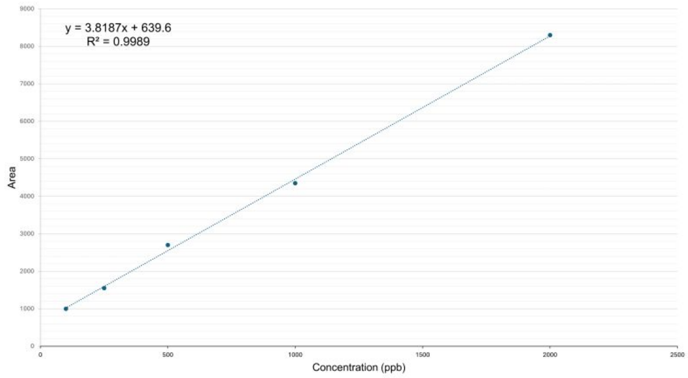

Figure S3. LC-MS calibration curve

1.3. Biphasic Gel Characterizations (BP Groups)

1.3.1. Rheological Measurements

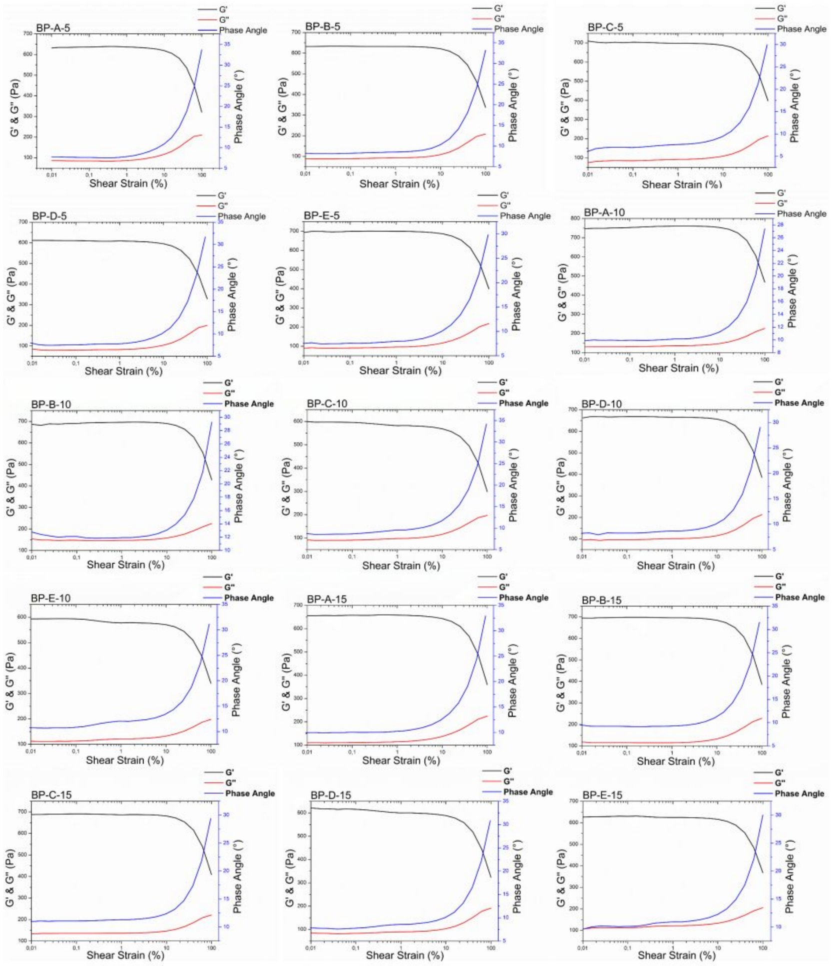

Figure S4. Rheology results of BP groups

According to the loss module results, a statistically significant difference was found between BP-A-5 and BP-A-10 (* P < 0.05), between BP-A-5 and BP-B-10 (** P < 0.01), between BP-B-5 and BP-B-10 (* P < 0.05), between BP-C-5 and BP-B-10 (* P < 0.05), BP-D-5 to BP-A-10 (* P < 0.05), BP-D-5 to BP-B-10 (** P < 0.01), BP-D-5 to BP-C-15 (* P < 0.05), BP-E-5 to BP-B-10 between (* P < 0.05), between BP-A-10 and BP-D-15 (* P < 0.05), between BP-B-10 and BP-C-10 (* P < 0.05) and between BP-B-10 and BP-D-15 (** P < 0.01).

According to the phase angle results, a statistically significant difference was found between BP-A-5 and BP-A-10 (* P < 0.05), between BP-A-5 and BP-B-10 (**** P < 0.0001), between BP-A-5 and BP-E-10 (*** P < 0.001), between BP-A-5 and BP-A-15. (* P < 0.05), between BP-A-5 and BP-C-15 (*** P < 0.001), between BP-A-5 and BP-E-15 (* P < 0.05), between BP-B-5 and BP-B-10 (*** P < 0.001), with BP-B-5 between BP-E-10 (* P < 0.05), between BP-B-5 and BP-C-15 (* P < 0.05), between BP-C-5 and BP-A-10 (** P < 0.01), between BP-C-5 and BP-B-10 (**** P < 0 .0001), between BP-C-5 and BP-E-10 (*** P < 0.001), between BP-C-5 and BP-A-15 (** P < 0.01), between BP-C-5 and BP-C-15 (**** P < 0.0001), between BP-C-5 and BP-E-15 between (** P < 0.01), between BP-D-5 and BP-A-10 (* P < 0.05), between BP-D-5 and BP-B-10 (**** P < 0.0001), between BP-D-5 and BP-E-10 (** P < 0 .01), between BP-D-5 and BP-A-15 (* P < 0.05), between BP-D-5 and BP-C-15 (*** P < 0.001), between BP-D-5 and BP-E-15 (* P < 0.05), between BP-E-5 and BP-A-10 (* P < 0.05), between BP-E-5 and BP-B-10 (**** P < 0.0001), between BP-E-5 and BP-E-10 (** P < 0.01), between BP-E-5 and BP-C-15 (** P < 0.01), BP-E-5 to BP-E-15 (* P < 0.05), BP-A-10 to BP-D-15 (* P < 0.05), BP-B-10 to BP-C-10 (*** P < 0.001), BP-B-10 to BP-D-10 (**** P < 0.0001), between BP-B-10 and BP-B-15 (** P < 0.01), between BP-B-10 and BP-D-15 (**** P < 0.0001), between BP-C-10 and BP-C-15 (* P < 0.05), between BP-D-10 and between BP-E-10 (* P < 0.05), between BP-D-10 and BP-C-15 (** P < 0.01), between BP-E-10 and BP-D-15 (** P < 0.01), between BP-C-15 and BP-D-15 (** P < 0.01), and between BP-D-15 and BP-E-15 (* P < 0.05).

1.3.2. Cohesivity Analysis

Table S2. Cohesivity of biphasic gels

Sample	Cohesivity

	(mg)
**BP-A-5**	391.33 ± 23.09
**BP-B-5**	425 ± 6.08
**BP-C-5**	393.33 ± 62.01
**BP-D-5**	406.67 ± 18.17
**BP-E-5**	421 ± 11.13
**BP-A-10**	385.67 ± 17.21
**BP-B-10**	391.33 ± 31.64
**BP-C-10**	422.67 ± 29.39
**BP-D-10**	450.33 ± 57.83
**BP-E-10**	359.67 ± 35.52
**BP-A-15**	441.67 ± 19.75
**BP-B-15**	470 ± 6
**BP-C-15**	415.5 ± 6.5
**BP-D-15**	480 ± 17
**BP-E-15**	488.33 ± 44.73

According to the cohesivity values, a statistically significant difference was found between BP-A-5 and BP-E-15 (* $P < 0.05$), between BP-C-5 and BP-E-15 (* $P < 0.05$), between BP-A-10 and BP-D-15 (* $P < 0.05$), between BP-A-10 and BP-E-15 (* $P < 0.05$), BP-B-10 to and BP-E-15 (* $P < 0.05$), BP-E-10 to and BP-B-15 (** $P < 0.01$), BP-E-10 to and BP-D-15 (** $P < 0.01$) and BP-E-10 and BP-E-15 (** $P < 0.01$).

1.3.3. Citric Acid Release Analysis

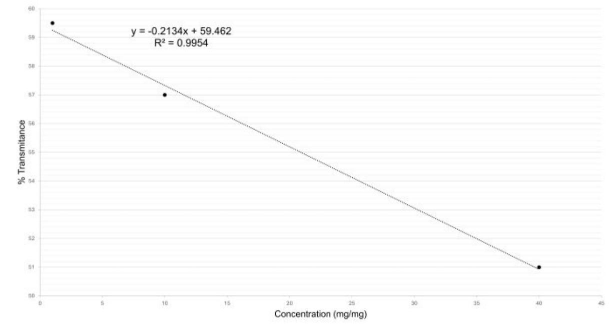

Figure S5. CA release calibration curve

1.4. Biphasic Gel Characterizations After Sterilization

1.4.1. Rheological Measurements

Table S3. Rheology results of final products after steam sterilization

Sample	Elastic Modulus (G', Pa)	Loss Modulus (G'', Pa)	Complex Viscosity (η*)	Phase Angle (°)
**BP-A-5-S**	151.56 ± 9.57	39.66 ± 7.21	4.98 ± 0.35	14.54 ± 1.68
**BP-B-5-S**	95.49 ± 30.6	18.22 ± 2.88	3.1 ± 0.93	12.51 ± 5.51
**BP-C-5-S**	201.75 ± 17.71	58.85 ± 2.18	6.69 ± 0.52	16.41 ± 1.91
**BP-D-5-S**	188.6 ± 5.44	63.8 ± 4.86	6.33 ± 0.21	18.66 ± 0.82
**BP-E-5-S**	103.91 ± 9.39	31.42 ± 1.68	3.45 ± 0.3	16.87 ± 0.59
**BP-A-10-S**	136.13 ± 15.74	35.7 ± 1.06	4.47 ± 0.49	14.88 ± 1.25
**BP-B-10-S**	286.58 ± 9.21	96.68 ± 3.34	9.62 ± 0.24	18.67 ± 1.12
**BP-C-10-S**	136.81 ± 20	36.47 ± 0.98	4.5 ± 0.6	15.41 ± 2.51
**BP-D-10-S**	176.73 ± 15.47	40.21 ± 1.83	5.76 ± 0.48	12.95 ± 1.15

**BP-E-10-S**	388.76 ± 13.91	88.89 ± 6.22	12.69 ± 0.47	12.86 ± 0.45
**BP-A-15-S**	168.58 ± 13.05	35.37 ± 1.32	5.48 ± 0.41	11.88 ± 0.46
**BP-B-15-S**	197.38 ± 13.1	41.34 ± 3.76	6.41 ± 0.43	11.81 ± 0.46
**BP-C-15-S**	141.67 ± 28.41	57.7 ± 3.05	4.88 ± 0.8	23.03 ± 5.14
**BP-D-15-S**	325.31 ± 10.26	140.83 ± 7.83	11.28 ± 0.4	23.41 ± 0.56
**BP-E-15-S**	150.3 ± 10.1	58.24 ± 4.35	5.13 ± 0.35	21.17 ± 0.15

Table S4. Change and % decrease in G' values before and after steam sterilization

Sample	Elastic Modulus (G', Pa)	Sample	Elastic Modulus (G', Pa)	% Decrease
**BP-A-5**	638.96 ± 54	**BP-A-5-S**	151.56 ± 9.57 (****)	76.28
**BP-B-5**	633.71 ± 107.05	**BP-B-5-S**	95.49 ± 30.6 (****)	84.93
**BP-C-5**	705.5 ± 119.05	**BP-C-5-S**	201.75 ± 17.71 (****)	71.40
**BP-D-5**	611.15 ± 80.75	**BP-D-5-S**	188.6 ± 5.44 (****)	69.14
**BP-E-5**	701.9 ± 144.91	**BP-E-5-S**	103.91 ± 9.39 (****)	85.19
**BP-A-10**	761.16 ± 158	**BP-A-10-S**	136.13 ± 15.74 (****)	82.11
**BP-B-10**	696.63 ± 154.05	**BP-B-10-S**	286.58 ± 9.21 (****)	58.86
**BP-C-10**	597.4 ± 64.1	**BP-C-10-S**	136.81 ± 20 (****)	77.09
**BP-D-10**	669.3 ± 47.3	**BP-D-10-S**	176.73 ± 15.47 (****)	73.59
**BP-E-10**	597.41 ± 98.93	**BP-E-10-S**	388.76 ± 13.91 (*)	34.92
**BP-A-15**	658.91 ± 168.5	**BP-A-15-S**	168.58 ± 13.05 (****)	74.41

**BP-B-15**	698.81 ± 97.85	**BP-B-15-S**	197.38 ± 13.1 (****)	71.75
**BP-C-15**	692.55 ± 117.45	**BP-C-15-S**	141.67 ± 28.41 (****)	79.54
**BP-D-15**	617.06 ± 42.31	**BP-D-15-S**	325.31 ± 10.26 (***)	47.28
**BP-E-15**	630.46 ± 98.1	**BP-E-15-S**	150.3 ± 10.1 (****)	76.16

References

[1] Ashwinkumar N, Maya S, Jayakumar R. Redox-responsive cystamine conjugated chitin–hyaluronic acid composite nanogels. RSC Adv 2014;4:49547–55. https://doi.org/10.1039/C4RA06578F.

[2] Yang Y, Zhao Y, Lan J, Kang Y, Zhang T, Ding Y, et al. Reduction-sensitive CD44 receptor-targeted hyaluronic acid derivative micelles for doxorubicin delivery. Int J Nanomedicine 2018;13:4361–78. https://doi.org/10.2147/IJN.S165359.

[3] Chen HY, Qin J, Hu Y. Efficient Degradation of High-Molecular-Weight Hyaluronic Acid by a Combination of Ultrasound, Hydrogen Peroxide, and Copper Ion. MOLECULES 2019;24. https://doi.org/10.3390/molecules24030617.

[4] Asiri AM, Al-Amoudi MS, Bazaid SA, Adam AA, Alamry KA, Anandan S. Enhanced visible light photodegradation of water pollutants over N-, S-doped titanium dioxide and n-titanium dioxide in the presence of inorganic anions. Journal of Saudi Chemical Society 2014;18:155–63. https://doi.org/https://doi.org/10.1016/j.jscs.2011.06.008.
